# Parametric Optimization and Effect of Nano-Graphene Mixed Dielectric Fluid on Performance of Wire Electrical Discharge Machining Process of Ni_55.8_Ti Shape Memory Alloy

**DOI:** 10.3390/ma14102533

**Published:** 2021-05-13

**Authors:** Rakesh Chaudhari, Jay Vora, L.N. López de Lacalle, Sakshum Khanna, Vivek K. Patel, Izaro Ayesta

**Affiliations:** 1 Department of Mechanical Engineering, School of Technology, Pandit Deendayal Energy University, Raisan, Gandhinagar 382007, India; chaudharirakesh5@gmail.com (R.C.); viveksaparia@gmail.com (V.K.P.); 2 Department of Mechanical Engineering, University of the Basque Country, Escuela Superior de Ingenieros Alameda de Urquijo s/n., 48013 Bilbao, Spain; izaro.ayesta@ehu.eus; 3 Department of Solar Energy, School of Technology, Pandit Deendayal Energy University, Raisan, Gandhinagar 382007, India; sakshum.khanna@gmail.com; 4 Journal of Visualized Experiments, New Delhi 110002, India

**Keywords:** nano-graphene powder, nitinol, shape memory alloy, WEDM, HTS algorithm

## Abstract

In the current scenario of manufacturing competitiveness, it is a requirement that new technologies are implemented in order to overcome the challenges of achieving component accuracy, high quality, acceptable surface finish, an increase in the production rate, and enhanced product life with a reduced environmental impact. Along with these conventional challenges, the machining of newly developed smart materials, such as shape memory alloys, also require inputs of intelligent machining strategies. Wire electrical discharge machining (WEDM) is one of the non-traditional machining methods which is independent of the mechanical properties of the work sample and is best suited for machining nitinol shape memory alloys. Nano powder-mixed dielectric fluid for the WEDM process is one of the ways of improving the process capabilities. In the current study, Taguchi’s L16 orthogonal array was implemented to perform the experiments. Current, pulse-on time, pulse-off time, and nano-graphene powder concentration were selected as input process parameters, with material removal rate (MRR) and surface roughness (SR) as output machining characteristics for investigations. The heat transfer search (HTS) algorithm was implemented for obtaining optimal combinations of input parameters for MRR and SR. Single objective optimization showed a maximum MRR of 1.55 mm^3^/s, and minimum SR of 2.68 µm. The Pareto curve was generated which gives the optimal non-dominant solutions.

## 1. Introduction

Shape memory alloys (SMAs) have started to become popular due to their unique ability of memorizing or regaining the original shape from the plastic deformed condition by means of heating or magnetic or mechanical loading. In 1932, SMA was first discovered Au-Cd by Arne Ölander [1]. Later on in 1941, Vernon and Vernon [2] described his polymeric dental material with the term “shape memory”. SMAs such as Fe-Mn-Si, Cu-Al-Ni, Co-Al, Zr-Cu, Cu-Zn, and Cu-Sn are some of the types of copper-based and iron-based SMAs, but poor thermo-mechanic performance, impracticability, and instability of these SMAs has restricted their applications in certain areas [3]. However, nickel-titanium based SMAs are more suitable for most of the applications, such as automotive sensors, air conditioning vents, structural elements, electronic cable connectors, valves, aerospace, actuators, oil industries, automobile, and robotics and MEMS devices [4,5]. These smart materials possess the main characteristic of Superelasticity (SE) and shape memory effect (SME) [6,7,8]. In addition to Pseudoelasticity and SME, nitinol is considered as an ideal material for biomedical applications owing to their properties such as biocompatibility, corrosion resistance, and wear resistance [9,10]. Machining of these newly developed smart materials require inputs of intelligent machining strategies. Conventional machining techniques for nitinol SMA possess several challenges such as formation of burr, poor chip breaking, high tool wear, and corrosion resistance [11,12]. This is due to high chemical reactivity, high toughness, high hardness, high ductility, and low strain hardening effect of nitinol SMA [13,14]. Wire electrical discharge machining (WEDM) is one of the non-traditional machining methods which is independent on mechanical properties of work sample, is best suited to overcome these defects [15]. All conductive materials can be machined through WEDM process. Non-contact operation of WEDM process between tool (wire) and workpiece significantly eliminates the problems of conventional machining process such as chatter, stress, and vibration [16]. WEDM process consists of multiple process variables which should be controlled to acquire great surface. Along with the better surface integrity, higher productivity is also key requirement of any industry that can be obtained by increasing MRR and simultaneously decreasing SR. Nano powder-mixed dielectric fluid for WEDM process is one of the ways of improving the process capabilities and achieving both the objectives simultaneously. The sparking frequency and thermal conductivity increases after adding powder concentration in dielectric fluid. It further increases the rate of erosion from work material [17]. Thus, this higher erosion rate also increases MRR. Insulating strength of dielectric fluid decreases after adding powder concentration in dielectric fluid. It further increases the inter-electrode gap condition [18]. Thus, this increase in inter-electrode gap condition improves the performance by obtaining superior surface finish. This realization leads to the initiation studies of Nano powder-mixed WEDM (NPMWEDM) of nitinol SMA.

WEDM technique consists of multiple input parameters and output responses. For multiple responses, conflicting situations arises between the selected responses. For example, current increases MRR considerably and, simultaneously, SR as well, which is not desirable. One approach to find solution for multi-objective optimization involving conflicting objectives is to convert them into a single-objective optimization by assigning weights to each objective function. However, the weighted approach cannot be considered as a global solution as the selection of the weights assigned to the objective function is dependent on designers and application, and it is susceptive to variations. In order to counter this, it has been proposed to probe a set of solutions rather than a single set which suffices the objective function. Moreover, these sets of solutions are non-dominated by other solutions and can hence be termed as non-dominated solutions. This non-dominated set of solutions is termed as the Pareto front. These fronts are basically a trade-off between two conflicting objectives. Such problems can be solved by using meta-heuristics-based optimization techniques. Researchers have developed various efficient advanced optimization techniques which have shown their effectiveness in optimizing process parameters for EDM processes. Heat transfer search (HTS) is one of such algorithm which is found to be easy to implement [19]. HTS algorithm was found to be useful for solving complex problems by finding global optimal solution [20,21].

Recently, powder-mixed dielectric fluid has turned out to be popular among the researchers to obtain optimum parametric setting for multiple objectives such as MRR and SR. Different powders such Si, W, Al, SiC, Cr, Ti, CNT, Al_2_O_3_, Gr/C, Mo, and Cu have been used by researchers which has been diluted with dielectric fluid for EDM process [18,22]. Important powder characteristics such as size of powder, concentration, thermal and electrical conductivity, and powder density have significant effect in process. Sahu and Mandal [23] studied the significance of graphite and alumina PMEDM process on surface integrity of nimonic 263 superalloy. Results shows improvement in surface defects for graphite powder mixed EDM process in comparison with other variants. MRR has been improved by 35% and machined surface shows reduction in micro-cracks by using graphite PMEDM process. Surekha et al. [24] studied the significance of aluminum PMEDM process on MRR and TWR of EN-19 alloy steel. Jeswani [25] explored the influence of 10 µm graphite powder for EDM process and his results shows an increased MRR by 60% and decreased TWR by 30%. Anil Kumar et al. [26] optimized silicon PMEDM process parameters of EN-24 tool steel. Results show that current, powder concentration, and pulse on time were observed to be the significant parameters of process. Sivaprakasam et al. [27] analyzed optimization of Nano-graphite PMEDM process of Inconel 718. Powder concentration of 0.5 g/L was found to be optimum for obtaining higher MRR and better surface finish. Mathapathi et al. [28] conducted their experiments with for graphite and Cr powder mixed with dielectric fluid. Experimental results showed increase in MRR value as powder concentration increases. MRR was also found to be largely influenced by peak current. Rathi et al. [29] examined the significance of various powders like graphite, aluminum oxide, and silicon to be mixed with dielectric fluid. Pulse-on time, Current, and duty cycle were considered as other input process parameters with the output machining characteristics of MRR and TWR. Bhiksha et al. [30] implemented Taguchi’s orthogonal array design to perform experiments in analyzing the significance of graphite PMEDM on MRR and SR. Results found that powder concentration and current mainly influences both objectives. Past studies on Nitinol SMA has shown retention of shape memory effect even after WEDM machining using Molybdenum wire as tool material [8,20]. Hence, the current study focuses more on the effect of Nano-graphene powder concentration on WEDM process parameters of Nitinol SMA and the parametric optimization of the selected responses. From the past literature, the effect of Nano-graphene powder mixed with dielectric fluid has not been explored properly for multi-objective optimization of machining variables of WEDM process. However, to the best of our knowledge, multi-objective optimization of NPMWEDM process parameters for Nitinol SMA has not yet been reported.

In the current study, current, pulse-on time, pulse-off time, and powder concentration has been identified as important machining variables while MRR and SR as the output parameters for PMWEDM process of Ni_55.8_Ti SMA. In the present study, Taguchi’s 4 level L16 orthogonal arrays have been used to perform the experiments. Adequacy and significance of process parameters was tested by ANOVA for each response variable. Mathematical models generated from regression analysis have been used for simultaneous optimization of output variables. In current study, advanced parameterless evolutionary HTS algorithm has been used to perform simultaneous optimization of response variables and case studies consisting of industrial requirements. Pareto curves have been generated using HTS algorithm which gives multiple optimal solution points. A validation study has been conducted to verify obtained results from algorithm. Lastly, investigation of machined surface was carried out using Scanning Electron Microscopy (SEM) to understand the effect of NPMWEDM process.

## 2. Synthesis of Nano-Graphene Powder

### 2.1. Reagents and Instrumentation

Natural graphite powder, 1, 2 Dichlorobenzene (DCB) and Ethyl alcohol (99.9%, *v*/*v*), was purchased from Sigma Aldrich Inc. All chemical reagents were analytical grade and used without further purification. The required equipment mainly included a Transmission electron microscope (TEM) (JEOL 2100, Tokyo, Japan), Field Emission-Scanning Electron Microscope (FESEM) (Zeiss Ultra 55, Bangalore, India), and Raman spectrometer (Renishaw in via Raman Microscope, Pune, India).

### 2.2. Synthesis of Graphene Using Carbon Source

In the process, 5 g of natural graphite was mixed with 1, 2 Dichlorobenzene (DCB) in a flask of 500 mL. The mixture was divided into 10 mL container and was ultrasonicated for 10 h. To avoid the heating and evaporation of the water due to heating, the water was changed frequently. After sonication, the sample was left unaltered for 48 h. Grey shade color dispersion was observed for the sample. Further, the colloidal dispersion was centrifuged at 5000 rpm for 15 min. Heavy lumps of non-reacted graphite lumps settled down and the dispersion is left with graphene sheet as a supernatant. The homogeneous dispersion was separated out in a separate vial and was again dispersed in ethanol solution. The above process was repeated 3–4 time; the finally centrifuged sample was filtered and dried in the vacuum (DP analytical, Gujarat, India) furnace to remove excess ethanol and DCB. The sonicated graphene sheets did not settle down even after few months and the morphological and structural properties were characterized using TEM, FESEM, and Raman spectrometer.

## 3. Materials and Methods

**Figure 1:**
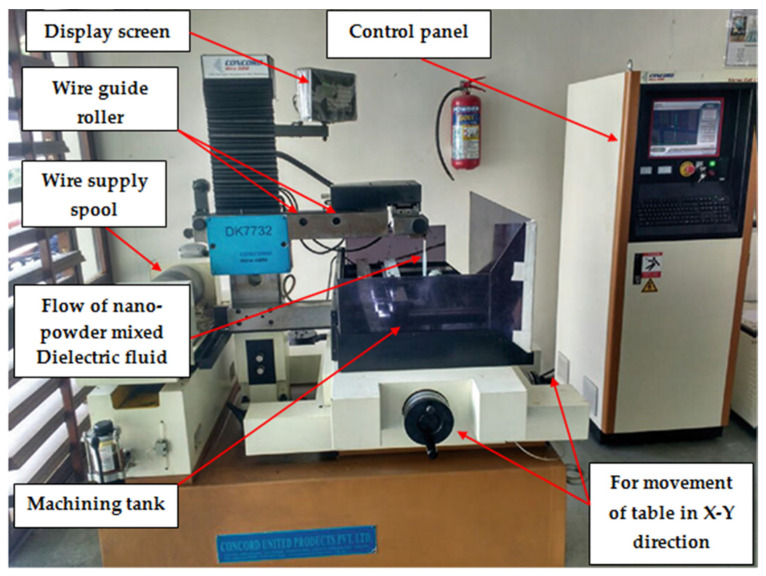
Experimental setup of WEDM process.

The Concord make DK7732 WEDM machine (Concord Limited, Bangalore, India) was used to perform the experiments of nitinol SMA. Nitinol rod of diameter 6 mm was procured from SMA wires, India and it is used as work material during WEDM process. Figure 1 show the experimental setup used in present study. Nano-graphene powder mixed dielectric fluid was sprayed through nozzles in the machined zone. Tool electrode (molybdenum wire) was selected which has a diameter of 0.18 mm. Table 1 shows the chemical composition of Nitinol SMA. Nano graphene powder was mixed with dielectric fluid in different concentrations while conducting the experiments. Selected levels of input process parameters (current, T_on_, T_off_, and powder concentration) were shown in Table 2. Input parameters along with their range were selected based on past literature and preliminary experimental trials. Taguchi’s 4 level L16 orthogonal arrays for 4 factors were used to design the experimental matrix, as shown in Table 3. Taguchi’s DOE approach has many benefits which make it favorable, particularly for experimental scientists. The main advantage of this technique is the reduction in the required number of experimental trials which incur cost, time, and resources for the investigation of significance of selected input variables parameters on output variables. Several input parameters of EDM based processes have been selected by different researchers. Taguchi method enables a large number of process parameters to be considered, and their effects on selected responses of the process to be analyzed.

To determine MRR, difference between weight of work sample before and after the machining was measured. MRR was measured in mm^3^/s as per the equation:

(1) $$\mathrm{MRR}\;=\frac{\Delta W*1000}{\rho *t}$$

where, ΔW = mass difference of workpiece between before and after the machining,

t = machining time to cut work sample in second,

ρ = density of workpiece (Nitinol SMA), 6.5 g/cm^3^

Mitutoyo make Surftest SJ-410 model surface roughness tester was used to evaluate SR of work material after machining, and the average of four values was considered as final SR value. Evaluation length of work surface and the cutoff length (λc) were selected as 5 mm and 0.8 mm, respectively. SR of the machined sample was recorded by measuring the arithmetic average-roughness (Ra) value. Scanning electron microscopy (SEM) has been used to investigate the surface morphology of the machined surface for conventional WEDM and NPMWEDM process.

## 4. Results and Discussions

### 4.1. Nano-Graphene Powder

**Figure 2:**
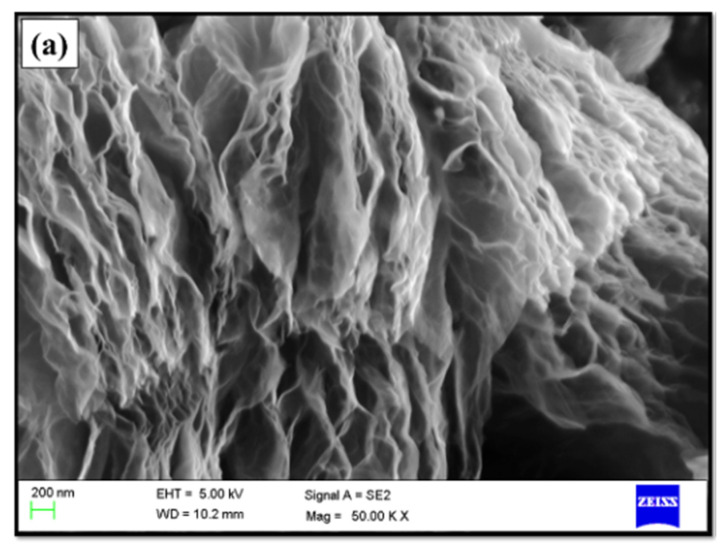

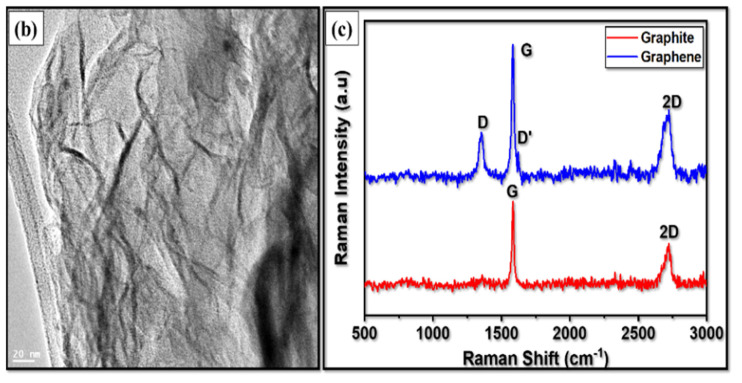
Morphological and structural characteristic of graphene (**a**) FESEM (**b**) TEM and (**c**) Raman profile.

The sonication technique creates a stress on the natural graphite flakes, which is transferred over the sp^2^ hybridized carbons present in the graphene layers and weakens the bond (van der Waals force) between the graphene layers which binds them as the stacks layers. The solvent dichlorobenzene helps in the development of sono-polymer, which act as an adhesive over and between the graphite layers. With increase in the time, polymer adhesion enhances and its binds over and within the graphite layers, which increases the distance between the layers. Thus, it leads to the synthesis of few layers of graphene by simple solvent-based sonication technique. The morphology of such graphene sheets were examined under FE-SEM and TEM as shown in Figure 2a,b. A 2D sheet structure of the exfoliated layers was observed with as sheet length of 400–600 nm. Further, Raman spectroscopy was performed to understand the structure properties and determine the number of graphene layers as shown in Figure 2c. The key Raman peaks D, G and 2D bands of graphene and graphite appeared at 1585 cm^−1^, 2710 cm^−1^ and 1354 cm^−1^, and 1585 cm^−1^ and 2720 cm^−1^, respectively. The key features of natural graphite and graphene is the presence 2D band. Therefore, the no. of graphene layers can be calculated from the position and shape of the 2D band. In comparison to natural graphite, a red shift of the 2D band was observed for graphene. The Raman spectra shown in Figure 2c shows a 2D band shape characteristic of few layer graphene sheet, along with some defects as shown by the D band. These defects can be due to the chemical exfoliation occurring during the sonication process [31]. Thus, the technique enables to produce large scale quality graphene using low cost technique.

### 4.2. Regression Equations

The measured values of responses, MRR and SR with their respective input machining variables were given in Table 3. The range of MRR value obtained for maximum to minimum is from 0.7410 to 0.1891 mm^3^/s, respectively, as per the sixteen conducted experiments. SR value from 4.12 to 6.52 µm was achieved. After analyzing the MRR and SR values, the mathematical correlation was generated for MRR and SR. Regression analysis was employed to generate mathematical relations among selected input process parameters of WEDM process and selected output response variables. Regression equations for output responses of MRR and SR are as shown in Equations (2) and (3):

(2) $$\mathrm{MRR}\;=-0.0730+0.0471\cdot \mathrm{Current}+0.0111\cdot T_{\mathrm{on}}-0.0143\cdot T_{\mathrm{off}\;}+0.1299\;\cdot \mathrm{Powder}\;\mathrm{Conc}.$$

(3) $$\mathrm{SR}\;=3.195+0.2522\;\cdot \mathrm{Current}+0.0565\cdot T_{\mathrm{on}}-0.0588\cdot T_{\mathrm{off}}-0.3550\;\cdot \mathrm{Powder}\;\mathrm{Conc}$$

Investigations of significance and non-significance of input variables was conducted by using ANOVA technique. Further, ANOVA was used for determining the percentage contribution of each input process parameter on the selected output response variables, for determination R-square values which signifies the data fitness, and for evaluation of standard deviation. Degree of freedom in ANOVA refers to the maximum number of observations in the data those are free to vary while estimating statistical parameters. DF is calculated by subtracting the number of relations from the number of observations. In present study, each variables considered in the equation is having 4 levels. As per the equation of DF (i.e., N-1, N = number of levels), DF for each factor comes out to be 3 and this calculated DF will further be used to calculate F and P statistics in ANOVA which gives the significance and non-significance of process parameters.

### 4.3. Analysis of MRR

Table 4 presents statistical analysis for MRR by using ANOVA technique. F and *p* value of ANOVA table gives the information about the significance/non-significance of input variable for selected response variable. At the 95% confidence interval, the value of P must be lower than 0.05 to signify a significant process parameter [32,33]. Table 4 of ANOVA for MRR shows that all the process parameters such as current, T_on_, T_off_, and powder concentrations are all significant as *p* value for all process parameters is less than 0.05. This means, all WEDM parameters are significantly affecting MRR value. The T_on_ was the most significant parameter contributing maximum 63.18%. The contributions from other parameters were T_off_ 17.09%, current 12.04% and the powder concentration of 7.09%. The error contribution was observed to negligible with 0.66%. This shows that the data in current study can be used for future predictions with least error. R-squared and adj R-squared values are observed to be 99.36% and 96.82%, respectively, for MRR. R-square value of 99.36% shows that present values can be useful for predicting 99.36% of future outcomes from this model. The selected model is considered to be best fit if difference of less than 20% is achieved between R-squared and Adj R-squared values [34]. For MRR, very less difference was obtained between both the values. Standard deviation of 0.0291282 has been obtained for MRR which means that maximum variation from the mean value is 0.0291282 for MRR.

**Figure 3:**
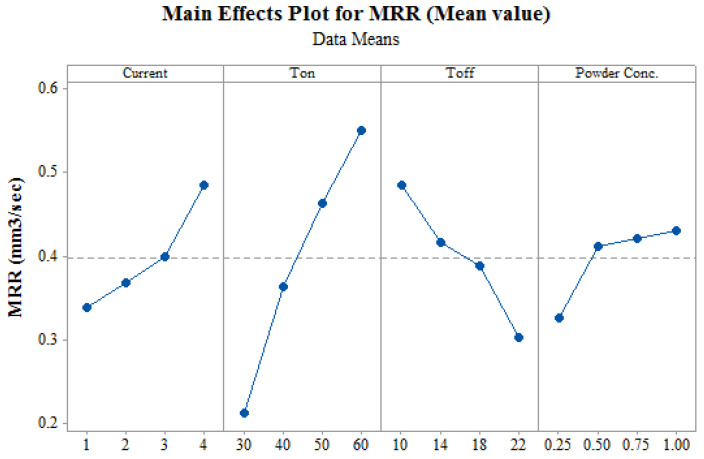
Effect of input process parameters on MRR.

Main effect plot for MRR which displays changes in MRR with respect to changes in input variables is presented in Figure 3. It has been observed that MRR is increased with an increase in current and T_on_. As T_on_ increases, it will further increase the generated discharge energy per spark [35]. In WEDM operation, discharge energy gets converted in thermal energy. The thermal energy then melts and vaporizes the material. So, higher the T_on_, higher will be discharge energy and which will then increase MRR value. Whereas an increase in current also increases discharge energy which then increases MRR value. Figure 3 shows the reverse effect of increase in T_off_ on MRR. Increase in T_off_ increases the time between two consecutive sparks which in turn decreases active sparks [15]. Pursuant to the same, lower discharge energy will be obtained at higher value of T_off_. So, as the T_off_ increases, MRR decreases due to reduction in spark thereby reducing the discharge energy. With the increase in Nano-powder concentration, increase in MRR was observed. The sparking frequency and thermal conductivity increases with the addition of Nano-powder in dielectric fluid which results in the increase of the erosion rate from the work surface [36]. Thus, this higher erosion rate also increases MRR. Dielectric field between wire and work material gets strengthened with the increase in Nano-powder in dielectric fluid as Nano-powder assists in bridging gap between tool and work material [27]. This improves deionization effect and thereby increases the rate of erosion. So, as Nano-powder concentration increases, the MRR increases, because of increase in erosion rate.

**Figure 4:**
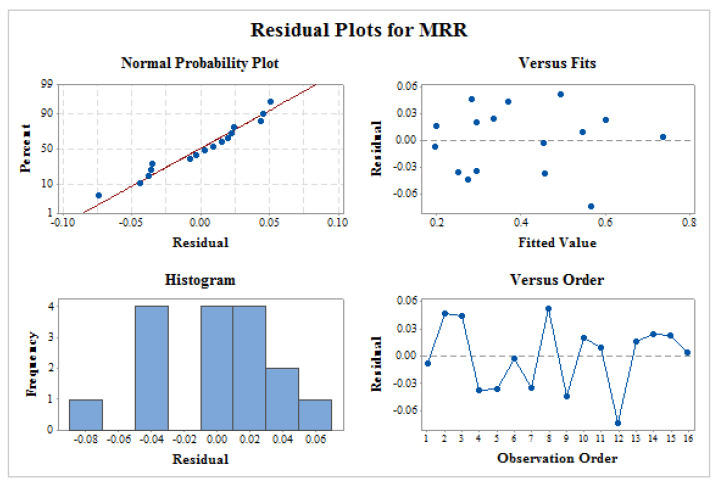
Residual plots for MRR.

Residual plot consisting of normal probability plot, fitted versus predicted pot, histogram plot, and time variance analysis was shown in Figure 4 for MRR. ANOVA results are considered to be valid depending on the analysis of these plots. Normality plot verifies that entire the residuals are on the straight line. This means all residuals are normally distributed and proposed mode is best suited. Residual versus fitted plot indicates the random allocation of residuals on both sided of reference line. This verifies a better statistical analysis of ANOVA. Histogram test shows a parabolic structure which is considered to be best solution for ANOVA and residual. This structure signifies good ANOVA results. No pattern was formed in the time variance plot which is the mandatory requirement of any significant ANOVA [20]. Therefore, all four tests of residual plot signify the ANOVA results for better future outcome of proposed model.

### 4.4. Analysis of SR

Statistical analysis for SR using ANOVA technique was shown in Table 5. At the 95% confidence interval, the value of P must be lower than 0.05 to signify a significant process parameter for selected output variable. Table 5 of ANOVA for SR shows that process parameters such as current, T_on_, and T_off_ are found to be significant while powder concentrations is non-significant effect on SR. The T_on_ was observed to be the most significant parameter contributing maximum 70.61% followed by T_off_ contribution of 13.66%, and current contribution of 13.50%. The error contribution was again observed to negligible with 0.65%. R-squared and adj R-squared values are observed to be 99.26% and 96.8%, respectively, for SR. The difference between R-squared and Adj R-squared values is very less which shows fitness of the model for selected responses. Standard deviation of 0.0141914 has been obtained for SR which means that maximum variation from the mean value is 0.141914 for SR.

**Figure 5:**
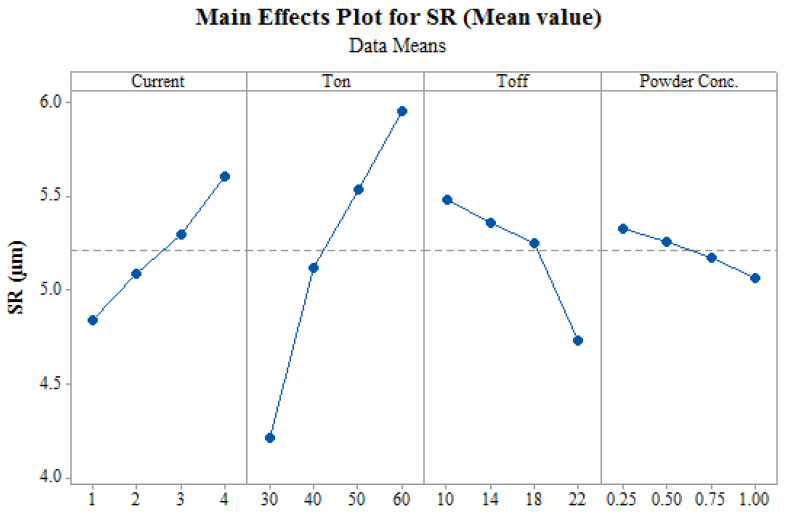
Effect of input process parameters on SR.

SR of the machined surface majorly dependent on the craters size which gets formed during the machining as material or debris gets eroded. Thermal energy which is formed by discharge energy is the main source for the debris formation. Increase in thermal energy forms larger and deeper craters on the machined surface and also deteriorate the machined surface. Pursuant to the same, machined surface becomes rough which in turn increases SR [8,37]. Figure 5 shows the variation of SR with input process parameter. It can be observed that current and T_on_ has the negative effect on surface quality of the machined surface. An increase in T_on_ also increases discharge energy which increases thermal energy. So, as T_on_ increases, the SR increases. At higher currents, the ionization of deionized water takes place, which leads to high discharge and thermal energy, creating larger and deeper craters, and increasing SR [38]. Figure 5 shows decrease in SR with increase in T_off_. Increased T_off_ decreases active sparks which results in decrease of the discharge energy. Lower discharge energy means lower thermal energy, and hence, small craters are formed which improves the SR of the machined surface. Figure 5 depicts that SR of machined surface decreases with the increase in Nano-powder concentration. Insulating strength of dielectric fluid decreases after adding powder concentration in dielectric fluid which in turn increases the conditions of inter-electrode gap [18]. Thus, this increase in inter-electrode gap condition improves the performance by obtaining superior surface finish [18]. Flushing of debris can be significantly improved by adding electrically conductive Nano-powder in dielectric fluid by increasing the discharge gap [39]. The addition of Nano-powder in dielectric fluid also enhances the sparking frequency and permits the uniform flushing of debris [40]. This results in formation of shallow craters which in turn provides the better surface finish. So, as Nano-powder concentration increases, the SR decreases, because of uniform sparking distribution and uniform flushing of debris. Thus, it was found that an addition/increase of Nano-powder in dielectric fluid significantly affects both the selected objectives of MRR and SR.

**Figure 6:**
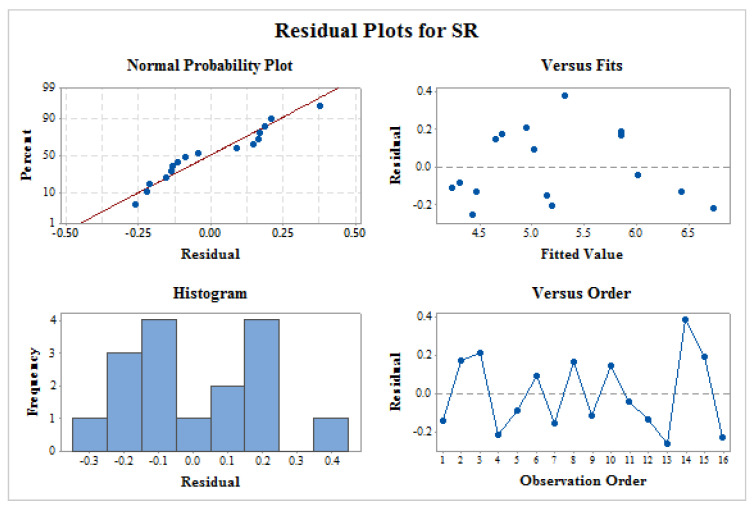
Residual plots for SR.

Residual plot consisting of normal probability plot, fitted versus predicted pot, histogram plot, and time variance analysis was shown in Figure 6 for SR. Similar patterns as that of MRR has been observed for all residual plots of SR. This shows that residual plot of SR also signifies the ANOVA results for better future outcome of proposed model.

### 4.5. Optimization Using HTS Algorithm

Patel and Savsani [19] proposed HTS algorithm based on the heat transfer principle which tries to reach thermal equilibrium. By means of transferring heat between system and surroundings, the algorithm reaches the equilibrium. For obtaining the thermal equilibrium condition, three heat transfer phenomenon namely conduction, convection and radiation are crucial. Transfer of heat is possible through any of these three modes of heat transfer ‘the conduction phase’, ‘the convection phase’, and ‘the radiation phase’. Random selection of any of the heat transfer phenomenon is selected in each generations. During the implementation, the system selects ‘n’ number of molecules which is nothing but the population size. Moreover, the temperature level known as design variables are selected randomly for each generations. This procedure gets repeated for next generation and population size gets updated by selection of suitable mode of heat transfer phenomenon. After getting the better value of function, the solution in the HTS algorithm gets accepted and worst solution get replaced by the elite solution in subsequent population.

#### 4.5.1. Conduction Phase

The solutions are updated in the conduction phase as per below Equations (4) and (5),

(4) Xj,i′={Xk,i+(−R2Xk,i), iff(Xj)>f(Xk)Xj,i+(−R2Xj,i), iff(Xj)<f(Xk);ifg≤gmaxCDF 

(5) Xj,i′={Xk,i+(−riXk,i), iff(Xj)>f(Xk)Xj,i+(−riXj,i), iff(Xj)<f(Xk);ifg>gmaxCDF

where, $X_{j,i}^{\prime }$ is the updated solution; j = 1,2,…,n; k is a randomly selected solution; j ≠ k; k ∈ (1, 2,…, n); i is a randomly selected design variable; i ∈ (1, 2,…, m); g_max_ is the maximum number of generation specified; CDF is the conduction factor; R is the probability variable; R ∈ {0, 0.3333}; r_i_ ∈ {0,1} is a uniformly distributed random number.

#### 4.5.2. Convection Phase

The solutions are updated in the convection phase as per below Equations (6) and (7),

(6) $$X_{j,i}^{\prime }=X_{j,i}+R\times \left(X_{s}-X_{\mathrm{ms}}\times \mathrm{TCF}\right)$$

(7) TCF={abs(R−ri), ifg≤gmaxCOFround(1+ri), ifg>gmaxCOF

where, $X_{j,i}^{\prime }$ is the updated solution; j = 1, 2,…, n; i = 1, 2,…, m. COF is the convection factor; R is the probability variable; R ∈ {0.6666, 1}; r_i_ ∈ {0, 1} is a uniformly distributed random number; X_s_ be the temperature of the surrounding and X_ms_ be the mean temperature of the system; TCF is a temperature change factor.

#### 4.5.3. Radiation Phase

The solutions are updated in the radiation phase as per below Equations (8) and (9),

(8) Xj,i′={Xj,i+R×(Xk,i−Xj,i), iff(Xj)>f(Xk)Xj,i+R×(Xj,i−Xk,i), iff(Xj)<f(Xk) ;ifg≤gmaxRDF

(9) Xj,i′={Xj,i+ri×(Xk,i−Xj,i), iff(Xj)>f(Xk)Xj,i+ri×(Xj,i−Xk,i), iff(Xj)<f(Xk) ;ifg>gmaxRDF

where, $X_{j,i}^{\prime }$ is the updated solution; j = 1, 2,…, n; i = 1, 2,…, m; j ≠ k; k ∈ (1, 2,…, n) and k is a randomly selected molecules; RDF is the radiation factor; R is the probability variable; R ∈ {0.3333, 0.6666}; r_i_ ∈ {0, 1} is a uniformly distributed random number.

The extreme machining limits of the used set-up were considered during the implementation of the algorithms.

T_on_: 1 µs ≤ T_on_ ≥ 110 µs

T_off_: 1 µs ≤ T_on_ ≥ 32 µs

Current: 1 A ≤ Current ≥ 6 A

Table 6 shows the results of single objective optimization for output responses of MRR and SR. Single objective optimization result shows that when any one objective is at optimal level, then the other objective is deviating from its optimal level. For example, for maximization of MRR, SR value was obtained as 10.51 µm which is far away from its optimal level. Pursuant to the same, for minimization of SR, MRR was obtained as 0.0001 mm^3^/s, which is too far from its optimal level. During single objective optimization of MRR and SR, levels of input variables are found to be contradictory. Such situation can be efficiently tackled by developing Pareto fronts with non-dominated optimum solutions. Pareto fronts, in essence, present a trade-off between two conflicting objectives, and manufacturers can select any point on the front.

**Figure 7:**
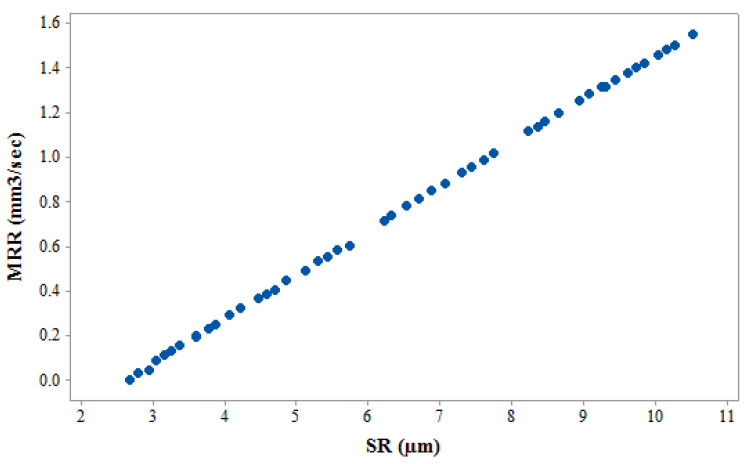
Pareto Graph of MRR vs. SR.

Simultaneous optimization of selected output variables (MRR and SR) was carried by implementing the multi-objective heat transfer search (MOHTS) algorithm. Non-dominant Pareto points were generated using MOHTS algorithm. Pareto graph of MRR and SR is shown in Figure 7. The X and Y axes of the Pareto curve stands for SR and MRR, respectively. 10,000 evolution functions has been used to obtain the desired Pareto points. 48 feasible Pareto points were shown in Figure 7. Table 7 shows these 48 feasible Pareto points along with the respective level of input process parameters. The conflicting nature of the graph can be clearly observed, as increase in MRR shows negative effect on SR. Every single Pareto point generated a distinctive solution. Pursuant to the same, operator has a choice to select the required Pareto point as per the required values of MRR and SR. Confirmation trials were conducted to verify the results obtained from MOHTS algorithm. Randomly, five experiments were selected from the obtained 48 feasible Pareto points (experiment numbers 1, 11, 23, 40, and 48) for validation. Table 8 shows the obtained experimental values of confirmatory trials along the predicted values of MOHTS algorithm. Table 8 shows negligible difference between the predicted and measured value. This shows the capability of developed model and HTS algorithm.

### 4.6. Effect of Nano-Graphene Powder on Response Variables

The effect of Nano-graphene powder concentration on selected response variables (MRR and SR) has been studied by comparing the results obtained between with and without the addition of Nano-graphene powder in dielectric fluid. To analyze these results, a case study of objective function (Equation (10)) was implemented as follows:

(10) $$\mathrm{Obj}\;=w_{1}\cdot \left(\mathrm{MRR}\right)+w_{2}\cdot \left(\mathrm{SR}\right)$$

An equal weight of 0.5 was assigned to both the response variables. The obtained values of MRR and SR for this present objective functions were 0.12187 mm^3^/s and 3.4945 µm, respectively, at the corresponding input parameters of current at 1 A, T_on_ at 30 µs, T_on_ at 22 µs, and powder concentration at 1 g/L.

To understand the effect of Nano-graphene powder on MRR and SR, another experiment was carried out without the addition of Nano-graphene powder in dielectric fluid. The obtained results are shown in Table 9. The MRR and SR values without the addition of Nano-graphene powder were obtained as 0.09051 mm^3^/s and 3.85 µm, respectively, at input parameters of current at 1 A, T_on_ at 30 µs, T_on_ at 22 µs, and powder concentration at 0 g/L. It can be observed from Table 9 that MRR and SR of the nitinol SMA are improved by 25.73% and 9.35%, respectively, with the addition of Nano-graphene powder concentration at 1 g/L.

### 4.7. Effect of Nano-Graphene Powder on Surface Morphology of Machined Surface

**Figure 8:**
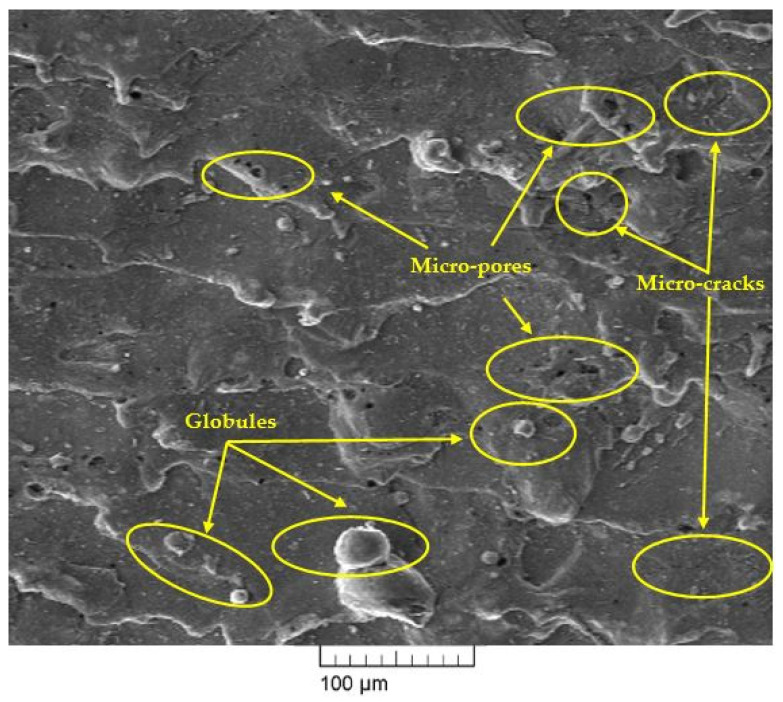
SEM micrograph of machined surface at Current = 1 A, T_on_ = 30 µs, T_off_ = 22 µs and Powder conc. = 0 g/L.

**Figure 9:**
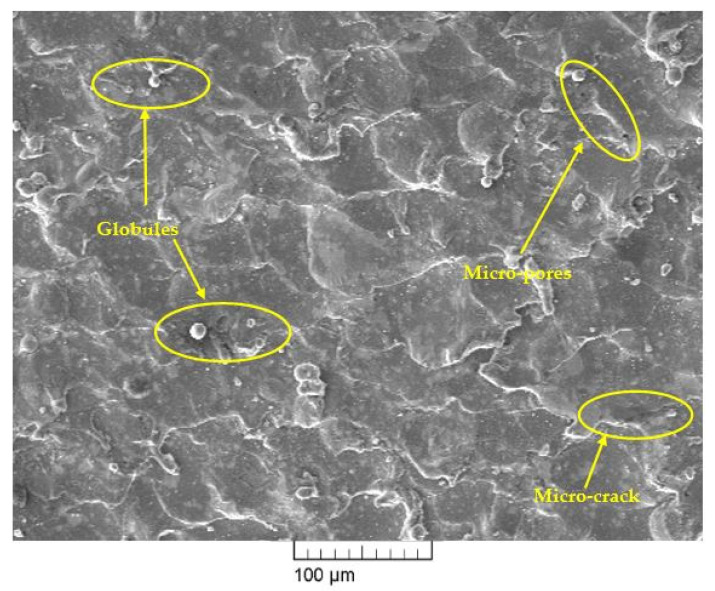
SEM micrograph of machined surface at Current = 1 A, T_on_ = 30 µs, T_off_ = 22 µs and Powder conc. = 1 g/L.

Scanning electron microscopy (SEM) has been used to investigate the surface morphology of the machined surface for conventional WEDM and NPMWEDM process. In this study, optimum corresponding process parameters shown in Table 9 has been used for the investigation of surface morphology of machined surface of conventional WEDM (current at 1 A, T_on_ at 30 µs, T_on_ at 22 µs, and powder concentration at 0 g/L) and NPMWEDM (current at 1 A, T_on_ at 30 µs, T_on_ at 22 µs, and powder concentration at 1 g/L) process. Figure 8 and Figure 9 shows the SEM micrographs of the machined surface at conventional WEDM and NPMWEDM process, respectively. By examining the surface obtained in Figure 8, it clearly shows the large presence of micro-pores, more deposition of layers, i.e., formation of globules and the presence of micro-cracks. However, machined surface of NPMWEDM which was obtained at same machining parameters as that of conventional WEDM process, shows a significant improvement in the surface defects, i.e., significant reduction in micro-pores, globules, and micro-cracks. Significant reduction in the micro-cracks of NPMWEDM process (Figure 9) is due to the uniform sparking between the work material and the tool [23,41]. The reason for large reduction in deposition of debris (globules) and micro-pores of NPMWEDM process is large gap between the tool and workpiece [42,43]. This leads to the appropriate and easy flushing of debris. This easy removal of debris forms small ridges resulting into improved surface quality.

## 5. Conclusions

In the current study, the effect of Nano-powder mixed WEDM performance of Nitinol SMA was presented. The following important conclusions can be drawn from this work:

All the input machining parameters such as current, pulse-on time, pulse-off time, and powder concentration have significant effect on MRR of Nano-powder mixed WEDM process. The T_on_ was the most significant parameter contributing maximum 63.18%. The contributions from other parameters were T_off_ 17.09%, current 12.04%, and the powder concentration of 7.09%.

The machining parameters current, pulse-on time, and pulse-off time have significance effect on SR of the machined surface. The T_on_ was observed to be the most significant parameter contributing maximum 70.61% followed by T_off_ contribution of 13.66%, and current contribution of 13.50%.

Increase in Nano-graphene powder concentration increases MRR due to higher erosion rate and simultaneously decreases SR due to uniform sparking distribution and uniform flushing of debris.

Generated regression models accurately predict MRR and SR values. Prediction capabilities of these models were confirmed by R-sq values and residual plot analysis.

The HTS algorithm was found to be very effective in predicting and optimizing both the response variables at different set of input process parameters.

A Pareto front was developed with non-dominated optimum solutions. Every single Pareto point gives a unique solution and has a corresponding value of input process parameter. Therefore, operator can select a suitable point by just observing their required values of MRR and SR.

MRR and SR of the nitinol SMA were improved by 24.01% and 9.35%, respectively, with the addition of Nano-graphene powder concentration at 1 g/L.

Surface morphology of machined surface has shown reduction in machining defects such as micro-pores, globules, and micro-cracks with the use of NPMWEDM process.

## Figures and Tables

**Table 1:** Chemical composition (wt.%) of Nitinol.

Element	Ti	Ni	Co	Cu	Cr	Fe	Nb	C	H	O	N
wt (%)	Balance	55.78	0.005	0.005	0.005	0.012	0.005	0.039	0.001	0.0344	0.001

**Table 2:** Nano-Powder Mixed WEDM conditions.

Working Condition	Description
Current (A)	1, 2, 3, 4
Pulse on time (µs)	30, 40, 50, 60
Pulse off time (µs)	10, 14, 18, 22
Powder concentration (g/L)	0.25, 0.50, 0.75, 1
Graphene nano powder-size (nm)	300–500
Powder	Graphite
Wire	Molybdenum

**Table 3:** Taguchi’s DOE with the measured values for MRR and SR.

Run	Current (A)	T_on_ (µs)	T_off_ (µs)	Powder Conc. (g/L)	MRR (mm^3^/s)	SR (µm)
1	1	30	10	0.25	0.1891	4.33
2	1	40	14	0.5	0.3293	4.88
3	1	50	18	0.75	0.4135	5.16
4	1	60	22	1	0.4184	4.98
5	2	30	14	0.75	0.2155	4.22
6	2	40	10	1	0.4502	5.11
7	2	50	22	0.25	0.2596	4.99
8	2	60	18	0.5	0.5472	6.02
9	3	30	18	1	0.2294	4.12
10	3	40	22	0.75	0.3147	4.8
11	3	50	10	0.5	0.5557	5.97
12	3	60	14	0.25	0.4940	6.3
13	4	30	22	0.5	0.2142	4.17
14	4	40	18	0.25	0.3592	5.7
15	4	50	14	1	0.6240	6.04
16	4	60	10	0.75	0.7410	6.52

**Table 4:** ANOVA for MRR.

Source	DF	SS	MS	F	P	Contribution (%)
Current	3	0.048154	0.016051	18.92	0.019	12.04
T_on_	3	0.252635	0.084212	99.25	0.002	63.18
T_off_	3	0.068354	0.022785	26.85	0.011	17.09
Powder Conc.	3	0.028141	0.009380	11.06	0.040	7.03
Error	3	0.002545	0.000848			0.66
Total	15	0.399831				
S = 0.02912, R-Sq = 99.36%, R-Sq (Adj) = 96.82%						

**Table 5:** ANOVA for SR.

Source	DF	SS	MS	F	P	Contribution (%)
Current	3	1.28002	0.42667	21.19	0.016	13.50
T_on_	3	6.68617	2.22872	110.66	0.001	70.51
T_off_	3	1.29577	0.43192	21.45	0.016	13.66
Powder Conc.	3	0.15937	0.05312	2.64	0.223	1.68
Error	3	0.06042	0.02014			0.65
Total	15	9.48174				
S = 0.141914, R-Sq = 99.26%, R-Sq (Adj) = 96.8%						

**Table 6:** Single Objective optimization results.

Objective Function	Design Variables				Objective Function	
	Current (A)	Pulse on Time (µs)	Pulse off Time (µs)	Powder Conc. (g/L)	MRR (mm^3^/s)	SR (µm)
Maximum MRR	6	110	1	1	1.5507	10.51
Minimum SR	1	1	8	1	0.0001	2.68

**Table 7:** Predicted results of HTS algorithm.

Sr. No.	Current (A)	Pulse on Time (µs)	Pulse off Time (µs)	Powder Conc. (g/L)	MRR (mm^3^/s)	SR (µm)
1	1	1	8	1	0.00013	2.67
2	1	1	6	1	0.02889	2.79
3	1	1	4	1	0.04363	2.95
4	1	1	2	1	0.08619	3.03
5	1	2	1	1	0.11150	3.14
6	1	6	3	1	0.12770	3.25
7	1	7	2	1	0.15313	3.37
8	1	12	3	1	0.19456	3.59
9	1	10	1	1	0.20095	3.59
10	1	14	2	1	0.23102	3.76
11	1	18	4	1	0.24660	3.87
12	1	18	1	1	0.28983	4.05
13	1	22	2	1	0.31991	4.21
14	2	23	3	1	0.36399	4.46
15	4	15	2	1	0.38370	4.57
16	4	17	2	1	0.40580	4.69
17	1	32	1	1	0.44609	4.84
18	3	29	2	1	0.49244	5.11
19	1	40	1	1	0.53524	5.29
20	2	40	3	1	0.55342	5.42
21	3	36	1	1	0.58469	5.57
22	2	45	3	1	0.60132	5.73
23	2	53	2	1	0.71254	6.22
24	1	58	1	1	0.73567	6.31
25	1	62	1	1	0.78033	6.53
26	1	65	1	1	0.81375	6.70
27	1	68	1	1	0.84708	6.87
28	2	68	2	1	0.87979	7.07
29	2	71	1	1	0.92738	7.30
30	1	78	1	1	0.95819	7.44
31	3	72	1	1	0.98581	7.60
32	2	79	1	1	1.01645	7.75
33	1	92	1	1	1.11416	8.23
34	4	81	1	1	1.13310	8.36
35	1	96	1	1	1.15895	8.46
36	2	95	1	1	1.19466	8.65
37	2	100	1	1	1.25053	8.93
38	1	107	1	1	1.28130	9.08
39	1	110	1	1	1.31493	9.25
40	3	103	2	1	1.31678	9.30
41	2	110	2	1	1.34733	9.44
42	4	103	1	1	1.37833	9.61
43	4	105	1	1	1.40027	9.72
44	4	107	1	1	1.42276	9.83
45	5	106	1	1	1.45891	10.03
46	5	108	1	1	1.48100	10.14
47	5	110	1	1	1.50357	10.26
48	6	110	1	1	1.55070	10.51

**Table 8:** Validation results for Pareto optimal points.

Sr. No.	Current (A)	Pulse on Time (µs)	Pulse off Time (µs)	Powder Conc. (g/L)	Predicted Values by HTS Algorithm		Experimentally Measured Values		% Deviation	
					MRR	SR	MRR	SR	MRR	SR
1	1	1	8	1	0.00013	2.67	0.00014	2.81	3.52	4.98
11	1	18	4	1	0.24660	3.87	0.24151	4.01	2.10	3.49
23	2	53	2	1	0.71254	6.22	0.73004	6.1	2.39	1.97
40	3	103	2	1	1.31678	9.30	1.35321	9.73	2.69	4.41
48	6	110	1	1	1.55070	10.51	1.49452	10.93	3.75	3.84

**Table 9:** Effect of Nano-graphene powder on MRR and SR.

Condition	Input Process Parameters	Response Variables
With addition of Nano-graphene powder at 1 g/L	Current = 1 A Pulse on time = 30 µs Pulse on time = 22 µs Powder conc. = 1 g/L	MRR = 0.12187 mm^3^/s SR = 3.4945 µm
Without Nano-graphene powder	Current = 1 A Pulse on time = 30 µs Pulse on time = 22 µs Powder conc. = 0 g/L	MRR = 0.09051 mm^3^/s SR = 3.85 µm

## Data Availability

The data presented in this study are available in this article.

## Nomenclature

ANOVA	Analysis of variance
DCB	Dichlorobenzene
DF	Degree of freedom
DOE	Design of Experiments
EDM	Electrical Discharge Machining
FESEM	Field emission scanning electron microscope
HTS	Heat transfer search
MOHTS	Multi-objective heat transfer search
MRR	Material removal rate (mm^3^/s)
NPMWEDM	Nano-powder mixed wire electrical discharge machining
PMEDM	Powder mixed electrical discharge machining
SEM	Scanning electron microscope
SMA	Shape memory alloy
SMAs	Shape memory alloys
SR	Surface roughness (µm)
TEM	Transmission electron microscope
T_on_	Pulse on time (µs)
T_off_	Pulse off time (µs)
WEDM	Wire electric discharge machine
